# Derivation of Breathing Metrics From a Photoplethysmogram at Rest: Machine Learning Methodology

**DOI:** 10.2196/13737

**Published:** 2020-07-31

**Authors:** Joseph Prinable, Peter Jones, David Boland, Cindy Thamrin, Alistair McEwan

**Affiliations:** 1 School of Electrical and Information Engineering The University of Sydney Darlington Australia; 2 The Woolcock Institute of Medical Research The University of Sydney Glebe Australia

**Keywords:** photoplethysmogram, respiration, asthma monitoring, LSTM

## Abstract

**Background:**

There has been a recent increased interest in monitoring health using wearable sensor technologies; however, few have focused on breathing. The ability to monitor breathing metrics may have indications both for general health as well as respiratory conditions such as asthma, where long-term monitoring of lung function has shown promising utility.

**Objective:**

In this paper, we explore a long short-term memory (LSTM) architecture and predict measures of interbreath intervals, respiratory rate, and the inspiration-expiration ratio from a photoplethysmogram signal. This serves as a proof-of-concept study of the applicability of a machine learning architecture to the derivation of respiratory metrics.

**Methods:**

A pulse oximeter was mounted to the left index finger of 9 healthy subjects who breathed at controlled respiratory rates. A respiratory band was used to collect a reference signal as a comparison.

**Results:**

Over a 40-second window, the LSTM model predicted a respiratory waveform through which breathing metrics could be derived with a bias value and 95% CI. Metrics included inspiration time (–0.16 seconds, –1.64 to 1.31 seconds), expiration time (0.09 seconds, –1.35 to 1.53 seconds), respiratory rate (0.12 breaths per minute, –2.13 to 2.37 breaths per minute), interbreath intervals (–0.07 seconds, –1.75 to 1.61 seconds), and the inspiration-expiration ratio (0.09, –0.66 to 0.84).

**Conclusions:**

A trained LSTM model shows acceptable accuracy for deriving breathing metrics and could be useful for long-term breathing monitoring in health. Its utility in respiratory disease (eg, asthma) warrants further investigation.

## Introduction

There has been increasing interest in monitoring health using wearable sensors. However, very few technologies have focused on the breathing signal. The ability to monitor breathing may be beneficial for general health and particularly for asthma, which is a health condition that affects over 300 million people globally [1]. Monitoring of lung function using specialized metrics such as peak expiratory flow has been shown to be useful for predicting risk of an asthma episode [2]; however, this can be difficult to perform for patients as it involves forced maneuvers. It remains to be seen whether continuous monitoring of simple breathing metrics such as the interbreath interval (IBI) and the inspiration-expiration (I:E) ratio could provide further information on asthma control [3] and disease status [4].

The availability of a noninvasive sensor that measures breathing continuously and in an ambulatory manner would facilitate studies to establish clinical utility. One sensor of interest is the pulse oximeter that is commonly used in a clinical setting to measure both arterial blood oxygen saturation (SPO_2_) and heart rate. A tidal breathing method exists that also shows promise for clinical prediction [5]; however, these methods are unsuitable for continuous monitoring (eg, during walking or exercise). It was recently shown that a pulse oximeter can also be used to continuously monitor respiratory rate in a clinical setting [6]. This is possible because breathing periodicity [6,7] and effort [8] modulate photoplethysmogram (PPG) amplitude, frequency, and baseline wander [9,10]. Filtering and feature-based signal processing approaches can be applied to the PPG signal to extract a surrogate respiratory signal. This in turn can be processed to derive breathing rate (BR) with varying degrees of accuracy [7].

Unfortunately, there is poor amplitude correlation between the surrogate respiratory waveform and a gold standard respiratory trace. This poor correlation may make the I:E ratio difficult or impossible to derive using existing methods. In this work, we sought to address this using machine learning. In a previous pilot study [11], we demonstrated how a long short-term memory (LSTM) approach could predict a respiratory waveform from which BR could be derived. LSTM is a type of a recurrent neural network that can capture long-term, time-based dependencies in data [12]. Through the LSTM, we showed that the Pearson correlation coefficient between the derived respiratory waveform and a pneumotachograph trace had similarly high *r* values (*r*>0.8) to existing methods. In this paper, we built on this study by investigating the accuracy to which IBI, I:E ratio, and BR respiratory metrics can be attained from a PPG-derived surrogate respiratory waveform using an LSTM. We show that, in comparison to existing approaches, we can derive breathing metrics to a higher degree of accuracy from a pulse oximeter.

## Methods

### Datasets

#### Data Collection

Measurements were recorded from a group of 10 healthy participants who provided informed consent. The protocol for this study was approved by Northern Sydney Local Health District Human Research Ethics Committee (LNR/16/HAWKE/99 ethics approval). Participants conducted 5 randomized breathing serials at a rate of 6, 8, 10, 12, or 14 breaths per minute (BPM). Each serial was conducted for 5 minutes. Each participant was coached to breath one full inhalation and exhalation in time with a visual prompt.

An Alice PDx (Philips Respironics, Murrysville, PA) portable sleep diagnostic system was used to measure physiological signals during this study. The supplied pulse oximeter was attached to the index finger of the nonmaster hand, allowing the capture of a raw PPG trace, SPO_2_, and pulse rate data. The Alice PDx reported calculated values for SPO_2_ and pulse rate 3 times per second. PPG signals were sampled at 75 Hz.

Respiratory inductance plethysmography is a method to measure relative tidal volume (RTV) as a function of the chest and abdominal wall movement [13]. In this study, inductance bands were placed around the abdomen and ribcage according to the manufacturer’s guidelines, allowing RTV to be estimated as the weighted sum of the chest and abdominal wall inductance signals. The Alice PDx system reported an RTV signal based on the contribution of both respiratory bands and was captured at 100 Hz.

#### Description of Available Features

The Alice PDx system outputs three independent time series: PPG, SPO_2_, and pulse rate. The SPO_2_, processed PPG, and pulse rate signals were up-sampled to 25 Hz while the RTV was down-sampled to 25 Hz before normalizing between ±1. The sampling rate of 25 Hz was selected to ensure respiratory rate accuracy [7,14] and so that all time series data had the same time scale.

In addition to the three time series given by the Alice PDx system, a bandpassed PPG time series was generated by passing the original PPG signal through a sixth order Butterworth bandpass filter with a center frequency corresponding to the respiratory rate of the signal with a bandwidth of 0.002 Hz. This additional time series was included because our previous findings suggested that this feature could improve model prediction [11].

Altogether, the available features used within our model are as follows:

- Feature 1: PPG
- Feature 2: bandpassed PPG
- Feature 3: SPO_2_
- Feature 4: pulse rate

We previously determined experimentally that the inclusion of SPO_2_ and pulse rate values helped inform the network when decoupling between the pulse signal and respiratory signal occurs [11]. The exact underlying physiological mechanisms are unclear.

### Derivation of a Respiratory Waveform Time Series

**Figure 1:**
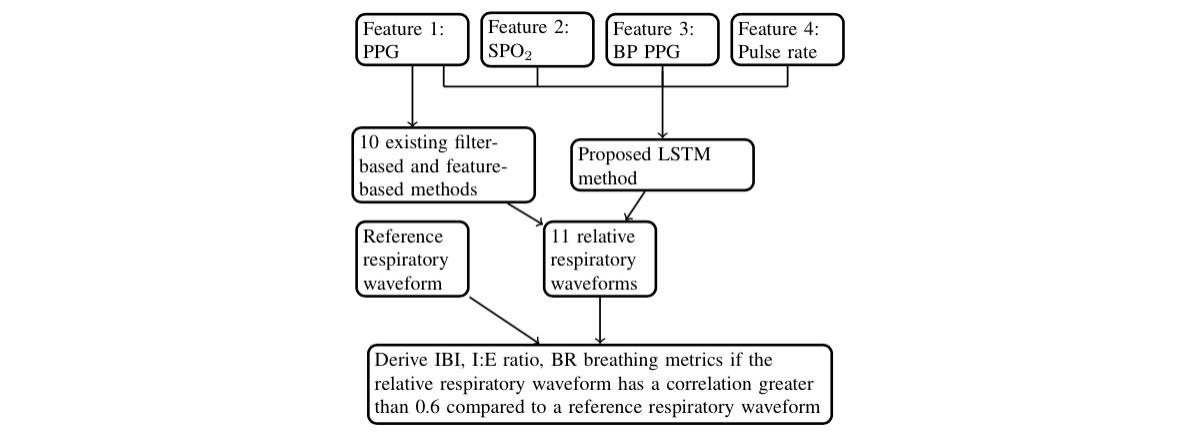
Using existing filter-based and feature-based methods, 10 relative respiratory waveforms were derived from a photoplethysmogram (PPG) signal, and another relative respiratory waveform was derived using a long short-term memory (LSTM) that accepts PPG, arterial blood oxygen saturation (SPO2), band-passed (BP) PPG, and pulse rate inputs. BR: breathing rate; I:E: inspiration-expiration ratio; IBI: interbreath interval.

For comparison purposes, RRest toolbox [15] was used to extract respiratory waveforms from a PPG using 10 feature-based and filter-based algorithms as shown in Figure 1. The resulting respiratory waveforms were temporally aligned to correspond with the reference respiratory waveform in the test set for comparison purposes. The techniques used to derive the respiratory waveforms, as well as our LSTM method, are described in Table 1.

**Table 1:** Techniques for the extraction of respiratory signals from a photoplethysmogram (adapted from Charlton et al [15]).

Respiratory signal		Description
**Filter-based**		
	X_A1_	Bandpass filter between plausible respiratory frequencies
	X_A2_	Maximum amplitude of the CWT^a^ within plausible cardiac frequencies (30-220 beats per minute) [16]
	X_A3_	The frequency corresponding to the maximum amplitude of the CWT within plausible cardiac frequencies [16]
**Feature-based**		
	X_B1_	Mean amplitude of troughs and proceeding peaks [7]
	X_B2_	Difference between the amplitudes of troughs and proceeding peaks [17]
	X_B3_	Time interval between consecutive troughs [17]
	X_B4_	Mean signal value between consecutive troughs [18]
	X_B5_	Peak amplitude [17]
	X_B6_	Trough amplitude [18]
	X_B10_	PPG^b^ pulse width estimation using a wave boundary detection algorithm [19]
**Machine learning–based**		
	X_LSTM_^c^	Proposed LSTM method

^a^CWT: continuous wavelet transform.

^b^PPG: photoplethysmogram.

^c^LSTM: long short-term memory.

### LSTM Architecture and Parameters

We propose the use of an LSTM model as an alternative to the signal processing methods described in Table 1. In this section, we discuss our training and validation procedures to determine an appropriate LSTM architecture to predict a respiratory waveform.

The core component of an LSTM architecture is a memory cell whose characteristics allow long-term data dependencies to be captured. A single LSTM cell uses gate mechanisms to forget irrelevant parts of a previous state, selectively update the current cell state, and to output the cell state [12]. Each cell contains a number of hidden units that define the dimensionality of both the current and output states. Increasing hidden units within a model may lead to overfitting. Conversely, reducing hidden units below a certain threshold will not allow a model to be trained.

#### Hyperparameter Search

We first conducted a structured, though nonexhaustive, hyperparameter search to determine suitable values for our final LSTM architecture. We then performed more extensive training to maximize the performance of our final architecture.

#### Hyperparameter Exploration

An open-source Python 3.5 library called TensorFlow r1.3 was used to train the LSTM model on a Dell Optiplex D810 (i7, 32 GB RAM; Dell Inc, Round Rock, TX) and two Titan Xp (Nvidia Corp, Santa Clara, CA) graphics processing units (GPUs).

The AdamOptimizer class of Tensorflow was used to train the LSTM using a learning rate of 0.0005 for 100 epochs with a batch size of 128. We explored the effect of changing the amount of cells (100, 300, 500), hidden units within a cell (500, 1500, 2500), and layers (1, 2, 3) and compared the results against a default model containing 100 cells, 500 hidden units, and a single layer. For this study, cells were layered sequentially two times to improve model accuracy and robustness [20]. The dropout layer was placed between each layer with a dropout rate of 0.5 to reduce overfitting [21]. There was a single dense, fully connected layer at the end.

To minimize training time for hyperparameter exploration, 4 smaller training datasets were created from the original 45 unique datasets (9 participants, each with 5 breathing serials). These datasets contained data from 1 participant (7), 3 participants (3, 5, 7), 5 participants (1, 3, 5, 7, 9), or 9 (1, 2, 3, 4, 5, 6, 7, 8, 9) participants. This allowed us to compare model performance as the number of participants increased for the various configurations. To further reduce training time, each dataset was reduced to 1 minute of data, splitting 70%, 15%, and 15% into training, validation, and test sets, respectively. To assess the performance of the model, we conducted 5-fold cross validation. To reduce computational time that typically results in higher error bias but lower variability, we chose 5 folds over 10 folds [22]. We investigated permutations of the available features and found that accuracy increased with the number of features with a minimal cost in terms of execution time.

**Figure 2:**
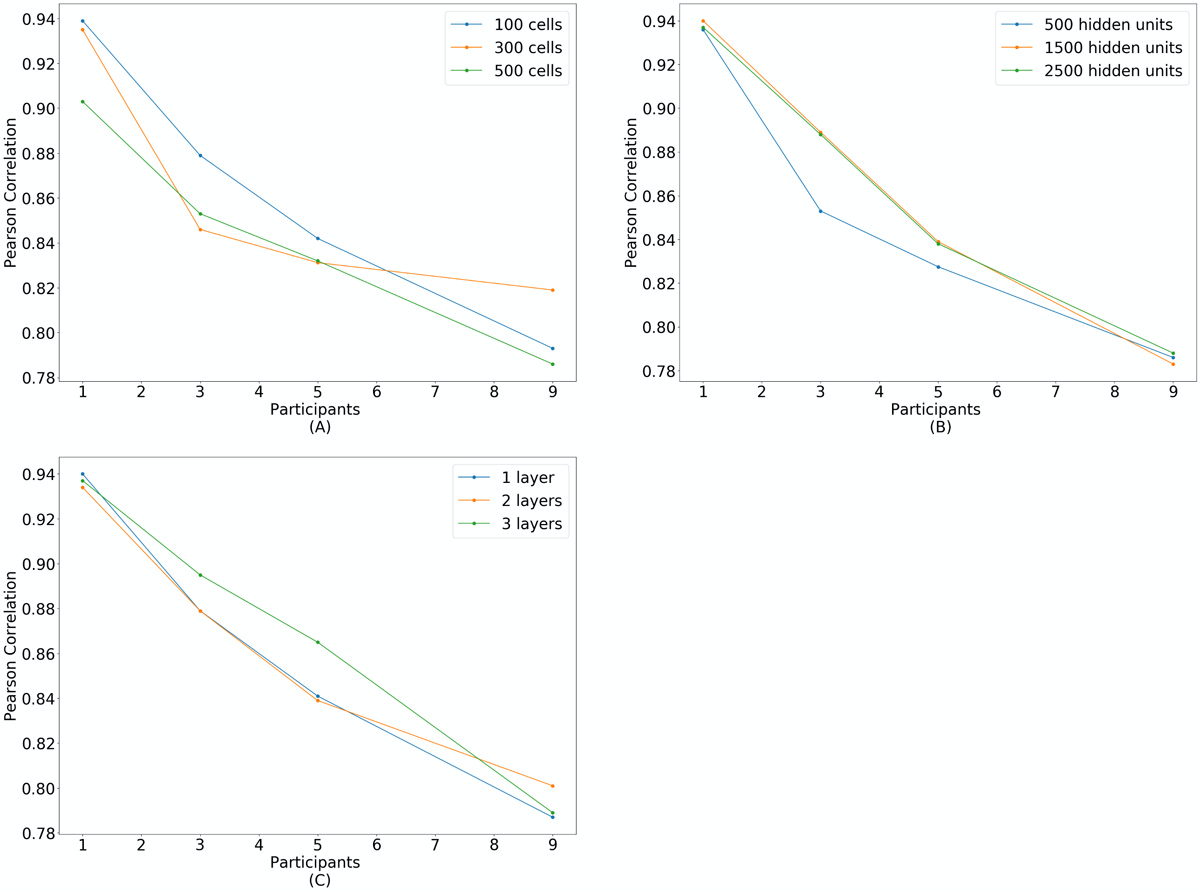
Pearson correlation values between derived and reference respiratory waveforms, given a dataset containing n participants, for a long short-term memory (LSTM) with (A) cells of size 100, 300, and 500; (B) hidden units of size 500, 1500, and 2500; (C) layers of size 1, 2, and 3.

Table 2 shows the training time in minutes as a function of participants and the hyperparameter. The Pearson correlation coefficients between the derived and reference respiratory waveforms are plotted as a function of increasing number of participants for the chosen cell values (Table 2) in Figure 2A, hidden unit values in Figure 2B, and layer values in Figure 2C. The highest correlation was achieved with 300 cells and 2 layers for 9 participants. For hidden units, the correlation was similar between the quantities, with 2500 hidden units only slightly better than 500 (0.786 vs 0.788). Due to the minimal difference, the latter was selected as it required significantly less training time (211 minutes vs 1213 minutes) for comparable performance.

**Table 2:** Training time (minutes) for the hyperparameter search.

Hyperparameters		1 participant		3 participants		5 participants		9 participants	
**Cells**									
	100		24		75		110		208
	300		54		200		272		505
	500		84		313		542		932
**Hidden units**									
	500		24		69		150		211
	1500		52		161		264		482
	2500		131		402		665		1213
**Layers**									
	1		24		65		116		220
	2		35		125		190		366
	3		48		158		271		470

### Final Model Training

The final model had around 8257 trainable parameters consisting of 300 cells, 2 layers, and 16 hidden layers. To train our final model, we used AdamOptimizer with an initial learning rate of 0.02 and batch size of 256. We conducted 5-fold cross validation with approximately 223,786 training examples per fold with early stopping.

### Extraction of Breathing Metrics

We defined a valid window when the Pearson correlation coefficient was >0.6 between the gold standard respiratory waveform and derived tidal volume waveform (TVW) in the window. For test sets that contained valid windows, we extracted peaks and troughs in MATLAB R2016b (MathWorks Inc, Natick, MA). To find the maximum points, ‘findpeaks’ was used, and we used a linear search algorithm to find the global minimum between 2 consecutive peaks. Using the peak and trough data, we extracted the following: IBI (the period in seconds between 2 consecutive peaks within the TVW signal), inspiration time (period in seconds between a trough and peak within the TVW signal), expiration period (period in seconds between a peak and trough within the TVW signal), and I:E (ratio between consecutive inspiration time and expiration period).

We then evaluated the Bland-Altman agreement [13] between the derived respiratory metrics to reference metrics.

Additionally, the root mean square error between hypothesized RTV signal y(t) and the true RTV Y(t) was calculated for each person and respiratory rate and subsequently averaged across the 5 folds.

## Results

### Data Collection

Data were acquired from 10 healthy subjects. One subject was excluded because of incomplete recordings due to an SD card save error on the Alice PDx. Therefore, data for 9 subjects were analyzed. The median (lower, upper quartiles) age of the analyzed subjects was 28 years (24.5 to 33.0 years). Median BMI was 23.59 kg/m^2^ (21.28 to 30.04 kg/m^2^), and 3 subjects (3/9, 33%) were female. In total, we recorded 3.75 hours of data, consisting of 5 minutes * 5 breathing rates * 9 participants.

### Model Validation

The weights and biases were saved for each epoch during training. Training was stopped when the validation error diverged to avoid overfitting. Early stopping occurred when the validation cost did not improve for 5 epochs.

### Derivation of Breathing Metrics

**Figure 3:**
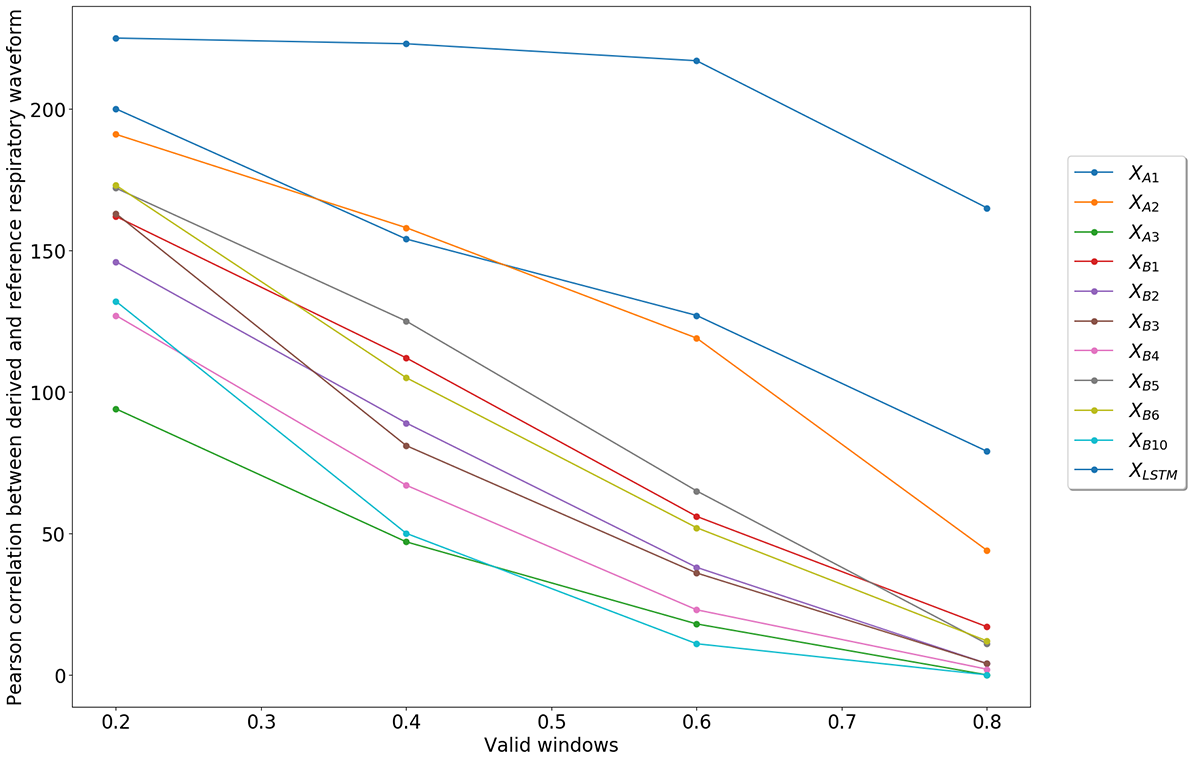
Number of valid windows as a function of increasing Pearson correlation coefficients between derived and reference respiratory waveforms in 0.2 increments. For an explanation of the variables please refer to Table 1.

In total, 225 unique test sets were created from 9 participants, at 5 respiratory rates, over 5 folds. Each test set was a window of 1000 samples (40 seconds) in length. We plotted the number of valid windows as a function of increasing Pearson correlation coefficients between derived and reference respiratory waveforms in 0.2 increments in Figure 3. For a Pearson correlation coefficient ≥0.6, our approach, X_LSTM_, was valid for 191/225 (85%) windows, while the next highest performing algorithm, X_A1_, was valid for 128/225 (57%) windows, followed by X_A2_, which was valid for 119/225 (53%) windows. Other algorithms were excluded from further analysis due to a small percentage of valid windows: 21/225 (9%) for X_A3_, 56/225 (25%) for X_B1_, 38/225 (17%) for X_B2_, 36/225 (16%) for X_B3_, 23/225 (10%) for X_B4_, 65/225 (29%) for X_B5_, 52/225 (23%) for X_B6_, and 11/225 (5%) for X_B10_.

Breathing metrics were averaged over each 40-second test set. The mean (SD) between derived and gold standard metrics and their associated *t* test results are shown in Table 3. The Bland-Altman agreement between derived and gold standard metrics for all subjects and respiratory rates are reported in Table 4. In the case of X_LSTM_, a Savitzky-Golay filter was used to smooth the derived respiratory waveform prior to extracting the breathing metrics.

**Figure 4:**
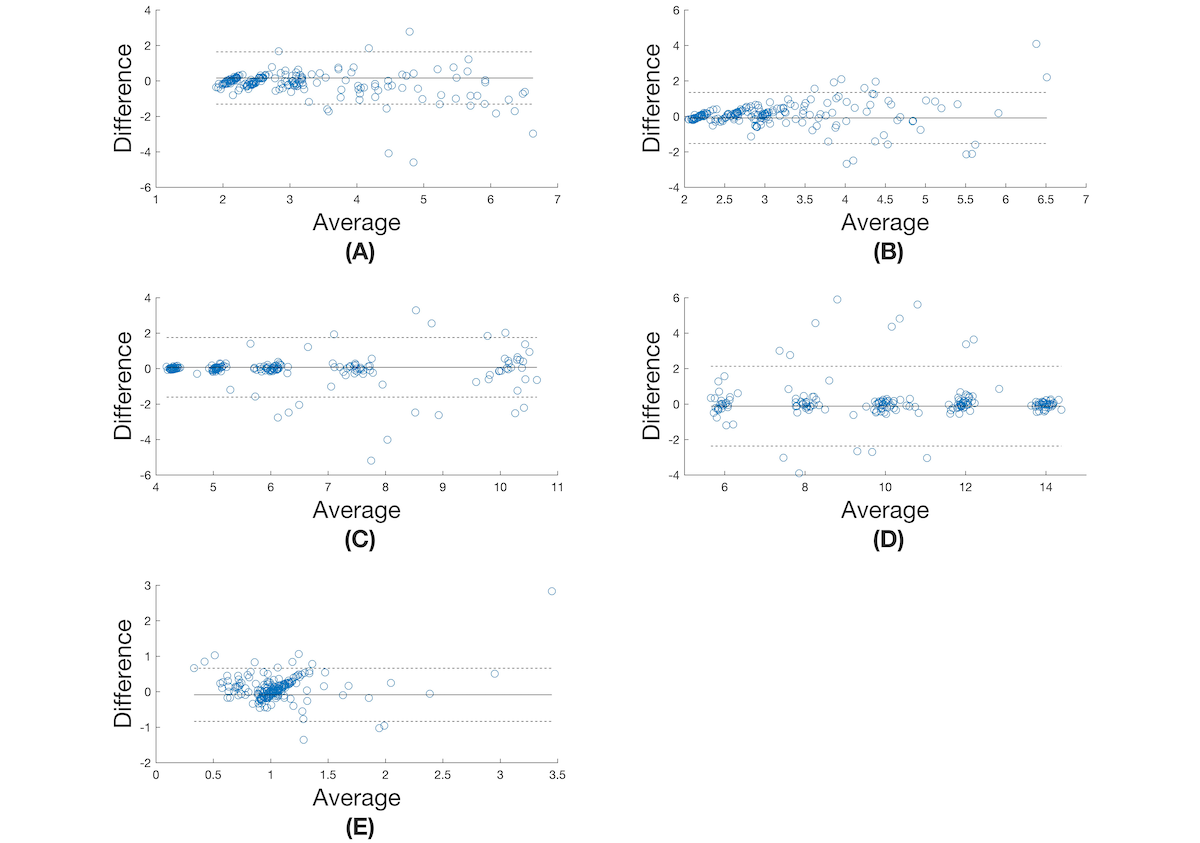
Bland-Altman plots for (A) inspiration time (seconds), (B) expiration time (seconds), (C) interbreath interval (seconds), (D) breathing rate (breaths per minute), and (E) inspiration:expiration ratio using the LSTM method.

**Figure 5:**
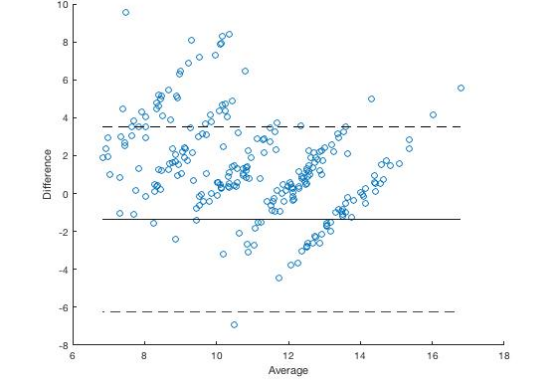
Bland-Altman plot for the highest performing algorithm (X_B1_,_2_,_3_E_T4_F_M1_) found by Charlton et al [7].

The Bland-Altman plot for the derived breathing metrics of inspiration time, expiration period, IBI, BR, and I:E across all participants (1-9) and all respiratory rates (6, 8, 10, 12, 14) using X_LSTM_ is shown in Figure 4. For comparison purposes, we report the Bland-Altman plot for derived respiratory rate across all participants and all respiratory rates using the highest performing algorithm found by Charlton et al [7] in Figure 5.

**Table 3:** Breathing metrics for the reference respiratory band, X_LSTM,_ X_A1_, and X_A2_ methods, with their associated paired t test results.

Breathing metrics	Respiratory band, mean (SD)	X_LSTM_^a^			X_A1_				X_A2_		
		Mean (SD)	t_180_ test	*P*	Mean (SD)	t_126_ test	*P*	Mean (SD)		t_118_ test	*P*
Tinsp^b^ (seconds)	3.28 (1.29)	3.14 (1.15)	2.92	0.004	3.46 (1.31)	1.65	0.103	3.40 (1.42)		0.02	0.99
Texp^c^ (seconds)	3.13 (1.01)	3.19 (1.05)	–1.68	0.095	3.38 (1.09)	–3.24	0.002	3.10 (0.95)		–1.44	0.152
BR^d^ (BPM^e^)	10.28 (2.72)	10.41 (2.74)	–1.39	0.167	9.69 (2.73)	1.93	0.056	10.35 (2.95)		–0.46	0.649
IBI^f^ (seconds)	6.40 (1.98)	6.33 (1.96)	1.12	0.262	6.84 (2.09)	–2.18	0.031	6.50 (2.09)		–1.73	0.086
I:E^g^	1.01 (0.36)	1.09 (0.43)	–3.09	0.002	1.03 (0.40)	–2.68	0.008	1.00 (0.29)		–1.50	0.135

^a^LSTM: long short-term memory.

^b^Tinsp: inspiration time.

^c^Texp: expiration period.

^d^BR: breathing rate.

^e^BPM: breaths per minute.

^f^IBI: interbreath interval.

^g^I:E: inspiration:expiration ratio.

**Table 4:** Derived breathing metrics using the X_LSTM_, X_A1_, and X_A2_ methods and associated statistical analyses.

Method		Bland-Altman r^2^	*P*	Absolute		Relative		
				Bias	95% LoA^a^		Bias (%)	95% LoA
**Tinsp (seconds) ^b^**								
	X_LSTM_^c^	0.70	<.001	–0.16	–1.64 to 1.31		–3.70	–38.44 to 31.05
	X_A1_	0.74	<.001	–0.11	–1.51 to 1.30		–2.35	–35.65 to 30.95
	X_A2_	0.74	<.001	-0.01	–1.46 to 1.46		–0.22	–33.34 to 32.90
**Texp (seconds)^d^**								
	X_LSTM_	0.54	<.001	0.09	–1.35 to 1.53		2.35	–31.84 to 36.55
	X_A1_	0.41	<.001	0.25	–1.45 to 1.95		6.41	–32.82 to 45.63
	X_A2_	0.43	<.001	0.10	–1.39 to 1.59		2.70	–36.34 to 41.73
**BR^e^** **(BPM^f^)**								
	X_LSTM_	0.83	<.001	0.12	–2.13 to 2.37		1.22	–23.63 to 26.07
	X_A1_	0.92	<.001	–0.13	–1.68 to 1.41		–1.38	–18.42 to 15.65
	X_A2_	0.88	<.001	0.04	–1.94 to 2.02		0.14	–19.35 to 19.62
**IBI^g^** **(seconds)**								
	X_LSTM_	0.82	<.001	–0.07	–1.75 to 1.61		–0.98	–22.62 to 20.66
	X_A1_	0.88	<.001	0.14	–1.31 to 1.60		2.08	–16.55 to 20.70
	X_A2_	0.91	<.001	0.10	–1.13 to 1.33		1.37	–16.20 to 18.94
**I:E^h^**								
	X_LSTM_	0.30	<.001	0.09	–0.66 to 0.84		9.91	–63.89 to 83.70
	X_A1_	0.11	<.001	0.09	–0.68 to 0.87		6.65	–61.43 to 74.73
	X_A2_	0.04	<.001	0.05	–0.62 to 0.71		3.41	63.89 to 70.72

^a^LoA: limits of agreement.

^b^Tinsp: inspiration time.

^c^LSTM: long short-term memory.

^d^Texp: expiration period.

^e^BR: breathing rate.

^f^BPM: breaths per minute.

^g^IBI: interbreath interval.

^h^I:E: inspiration:expiration ratio.

Our model consistently performed comparably to the other methods, showing similar agreement (lower bias) and variability (narrower limits of agreement). The relative bias for our model was <4% for all breathing metrics examined except for I:E ratio (at 9.9%), which is within the limits of accuracy of existing standards on the estimation of breathing metrics using conventional methods [5], although the limits of variability are wide.

The differences for inspiration time are bound within the 95% CIs for average inspiration periods <4 seconds. Distinct clustering can be seen around an inspiration period of 2 seconds (Figure 4A). The differences for expiration period are bound within the 95% CIs for average expiration periods of 2-3 seconds (Figure 4B). For IBI, 4 distinct clusters occur corresponding to intervals of 4, 5, 6, and 7 seconds; however, the clustering weakens above 9 seconds (Figure 4C). For BR, 5 distinct clusters are formed corresponding to expected BRs of 6, 8, 10, 12, and 14 BPM (Figure 4D). There is noticeable clustering for I:Es of 0.8-1 (Figure 4E).

To quantify the accuracy of our model and provide a metric for future comparisons, we report the root mean square error over all participants and respiratory rates for X_LSTM_ for inspiration time (0.77 seconds), expiration period (0.74 seconds), IBI (0.8377 seconds), BR (0.86 BPM), and I:E (1.15).

## Discussion

### Principal Findings

In this work, we were interested in determining the feasibility of finding continuous measures of inspiration time, expiration period, IBI, BR, and I:E metrics from a PPG. We showed how an LSTM architecture could be used to predict these metrics for 191/225 (85%) test sets comprised of 9 participants at a respiratory rate of 6, 8, 10, 12, or 14 BPM. We conducted Bland-Altman analyses and found the LSTM was able to predict the average inspiration time of –0.16 seconds (–1.64 to 1.31 seconds) and expiration period of 0.09 seconds (–1.35 to 1.53 seconds) over a 40-second window. The LSTM was able to predict an I:E ratio of 0.09 (–0.66 to 0.84), although this was poorly correlated with reference values. However, this is the first time this metric is being reported in the literature as measured from a pulse signal.

The LSTM model was trained to minimize the error between derived and reference respiratory waveforms and was then able to generalize the breathing characteristics of 9 subjects and predict future respiratory waveforms based on PPG data. The ability to “see and learn” a reference signal presents a distinct advantage over existing methods. Through this approach, it was possible to determine the continuous average breathing metrics of inspiration time, expiration period, IBI, and BR for the majority of time (85%), exceeding a Pearson correlation threshold of 0.6. In contrast, these breathing metrics could only be derived, at best, around half the time (56% in the case of X_A1_) using existing feature-based and filter-based algorithms that did not rely on any previous reference data. While we directly compared the performance of X_A1_ and X_A2_ to the LSTM method, other methods were excluded from this analysis due to the fact that the correlation between the derived respiratory waveform and the gold standard was <0.6 more than 80% of the time. Feature-based techniques (X_B1_-X_B6_, X_B10_) have performed well in previous respiratory rate algorithm assessments by Charlton et al [7] and would likely have similar performance on this dataset. In the cases where breathing metrics could be extracted for X_A1_and X_A2_, we found that the metrics of inspiration time, expiration period, and I:E were poorly correlated with the reference metrics, as shown in Table 4.

We conducted Bland-Altman analysis on the highest performing algorithm X_B1,2,3_E_T4_F_M1_ found by Charlton et al [7] in his comparison of classical signal processing algorithms for PPG. The bias in our dataset compared to those in the dataset used by Charlton et al [7] was higher (–1.12 vs 1). However, the 95% limits of agreement (BPM) was lower (–2.4 to 2.1 vs –5.1 to 7.2). X_LSTM_ compares favorably to X_B1,2,3_E_T4_F_M1_ with similar bias (0.12 vs –1.10) and a smaller 95% limits of agreement (BPM; –2.13 to 2.37 vs –2.63 to 2.44). The bias in our model compares well against existing standards on breathing metric estimation using conventional methods, which stipulate an accuracy of at least 2% for respiratory rate. It is worth noting that the standards are formulated for infant populations who breath faster. The wide variability seen in our model could be improved, although it is lower than that obtained from other methods examined. The high degree of variability could arise from differences in accuracy with different respiratory rates. While there is insufficient data from this study to ascertain this, it justifies use of longer-term data collection for further investigation.

The hyperparameters for the LSTM model were chosen in a structured, although non-exhaustive, manner by comparing a change in the number of cells, hidden units, or layers to a fixed model. Figures 2-4 show a decreasing trend in the correlation between the LSTM-derived respiratory waveform and the reference waveform as the number of participants increased. This trend occurred irrespective of the number of cells, hidden units, or layers. This may be accounted for, in part, by the complexity for which the LSTM model must account as the participant population increases. In the specific case of 300 cells, the correlation curve decreased quasi-exponentially. However, in the case of hidden units and layers, the correlation curve decreased quasi-linearly. It remains to be seen if the minimum correlation is bound between derived and reference respiratory waveforms for a given population. The findings of this paper show that our previous network parameter was much larger than required [11].

In this work, we used the following 4 features: PPG, filtered PPG, SPO_2_, and pulse rate. We did not conduct feature selection, which may have helped to improve the overall model performance. It would be useful to see the effect of removing the filtered PPG signal feature to reduce additional preprocessing time and computational power.

Due to a limited participant population, we did not conduct leave-one-out participant cross validation. The shape of each respiratory waveform varied from person to person, and it is unlikely that the LSTM model derived in this work would be able to predict respiratory metrics from an unseen participant. However, with a larger training population, the LSTM model may be exposed to enough data to enable the accurate prediction of respiratory metrics in an unseen participant. Previously, we found that participants would prefer that a wearable sensor device have a watch form factor [23]. In this paper, we did not look at the feasibility of implementing an LSTM in this type of form factor. Currently, LSTM training requires GPU-grade computational power. With current low-power Bluetooth low energy devices [11,24,25], it may be possible to acquire PPG data and stream real-time data to a cloud-based GPU server to run online training. Once the weights and biases of the LSTM architecture are found, it may also be possible for an embedded platform to perform the required processing to obtain real-time breathing metric predictions. At present, field-programmable gate arrays can be used for real-time predictions and benefit from low latency and low power consumption [26]. Additionally, the field-programmable gate array architecture is reconfigurable. This would allow any potential device to be individually tailored to a specific model.

### Conclusion

This paper presents the feasibility of monitoring simple breathing metrics such as the IBI, BR, inspiration time, expiration period, and I:E for a person at rest. We hope this proof-of-concept paper will inspire future research to collect further data and develop more powerful machine learning algorithms. In the future, it may also be possible to derive these metrics from a wristworn device that contains a pulse oximeter and accelerometer for a person at rest and support potential longitudinal studies to determine if these metrics can provide further information on asthma type [3] and provide any clinical utility [4].

## Abbreviations

BPM: breaths per minute.
BR: breathing rate.
I:E: inspiration:expiration ratio.
IBI: interbreath interval.
GPU: graphics processing unit.
LoA: limits of agreement
LSTM: long short-term memory.
PPG: photoplethysmogram.
RTV: relative tidal volume.
Texp: expiration period.
Tinsp: inspiration time.
TVW: tidal volume waveform.
